# The mid-domain effect in flowering phenology

**DOI:** 10.1016/j.pld.2024.05.005

**Published:** 2024-05-25

**Authors:** Yanjun Du, Rongchen Zhang, Xinran Tang, Xinyang Wang, Lingfeng Mao, Guoke Chen, Jiangshan Lai, Keping Ma

**Affiliations:** a School of Tropical Agriculture and Forestry (School of Agricultural and Rural Affairs, School of Rural Revitalization), Hainan University, Haikou 570228, China; b HNU-ASU Joint International Tourism College, Hainan University, Haikou 570228, China; c School of Ecological and Environmental Sciences, East China Normal University, Shanghai 200062, China; d College of Ecology and Environment, Nanjing Forestry University, Nanjing 210037, China; e State Key Laboratory of Vegetation and Environmental Change, Institute of Botany, Chinese Academy of Sciences, Beijing 100093, China

**Keywords:** Flowering diversity, Functional biogeography, Latitudinal gradient, Macroecology, Macrophenology, Null model

## Abstract

The timing of flowering is an important driver of species distribution and community assembly patterns. However, we still have much to learn about the factors that shape flowering diversity (i.e., number of species flowering per period) in plant communities. One potential explanation of flowering diversity is the mid-domain effect, which states that geometric constraints on species ranges within a bounded domain (space or time) will yield a mid-domain peak in diversity regardless of ecological factors. Here, we determine whether the mid-domain effect explains peak flowering time (i.e., when most species of communities are flowering) across China. We used phenological data of 16,267 herbaceous and woody species from the provincial *Flora* in China and species distribution data from the Chinese Vascular Plant Distribution Database to determine relationships between the observed number of species flowering and the number of species flowering as predicted by the mid-domain effect model, as well as between three climatic variables (mean minimum monthly temperature, mean monthly precipitation, and mean monthly sunshine duration). We found that the mid-domain effect explained a significant proportion of the temporal variation in flowering diversity across all species in China. Further, the mid-domain effect explained a greater proportion of variance in flowering diversity at higher latitudes than at lower latitudes. The patterns of flowering diversity for both herbaceous and woody species were related to both the mid-domain effect and environmental variables. Our findings indicate that including geometric constraints in conjunction with abiotic and biotic predictors will improve predictions of flowering diversity patterns.

## Highlights

• The mid-domain effect explains a significant proportion of the temporal variation in flowering diversity.
• The mid-domain effect explains more variance in flowering diversity at higher than at lower latitudes.
• The predictive power of the mid-domain effect is stronger than that of climatic variables.
• Geometric constraints are of sufficient magnitude to warrant further consideration as drivers of phenology.

## 1 Introduction

The seasonal timing of flowering underpins several aspects of community assembly and ecosystem function (Post et al., 2001; Cleland et al., 2006). Although some plant species flower throughout the growing season (Fenner, 1998), in most plant communities, flowering of most plant species overlaps at a specific point in time, which is referred to as the peak flowering time. Peak flowering times are important because their patterns often involve the co-evolved behaviors of plants and pollinators, which may contribute to community assembly in ways involving species diversity and dominance patterns (Heithaus, 1974; Rathcke and Lacey, 1985). Thus, shifts in peak flowering times have broad implications for ecological interactions, including attraction of mutualists, avoidance of antagonists, and plant–plant competition (via changes in seedling density and emergence times), which could thus reshape plant communities (Wright, 1996; Thies and Kalko, 2004; Pau et al., 2011; CaraDonna et al., 2014). Although we know flowering times of individual species are affected by environmental factors such as temperature, precipitation, and irradiance (Frankie et al., 1974; Reich and Borchert, 1984; Wright and Vanschaik, 1994), we still have much to learn about the forces that shape peak flowering time at the community scale.

The mid-domain hypothesis, which was introduced to help explain latitudinal gradients in species diversity (Colwell and Hurtt, 1994; Colwell and Lees, 2000), predicts that if ranges of species are randomly placed within a bounded domain, the greatest overlap of ranges, and thus species richness, will occur at the center of the domain (Colwell and Hurtt, 1994). This mid-domain effect has been used to explain latitudinal and elevational gradients of species richness in both plants (Colwell et al., 2004) and animals (Chen et al., 2020; Chettri and Acharya, 2020; Siqueira et al., 2021). Mid-domain effects have also been proposed for temporal gradients, whereby randomly distributed flowering periods will tend to overlap most frequently at the mid-domain, which is in this case during the middle of the growing season (Morales et al., 2005; Letten et al., 2013). If flowering time is neutral within a growing season and is uninfluenced by species interactions and environmental conditions, then a null model such as the mid-domain hypothesis may accurately predict patterns of flowering phenology at the community level. The premise of this mid-domain effect in flowering phenology is that the onset of the growing period constrains the timing of flowering in most species, with its end setting a hard boundary for fruiting (and indirectly, flowering) because seed production must be completed before plant growth ceases (Morin and Chuine, 2006). One of the few existing studies that has tested the mid-domain effect in flowering diversity (number of species flowering per period) found that the “mid-domain” null model qualitatively matched the pattern of flowering species richness in a sub-alpine forb community in Colorado, USA (Morales et al., 2005).

Previous studies have indicated that phenological diversity (i.e., the number of species flowering per month) may be affected by latitude and growth habit. At higher latitudes, plant assemblages such as temperate forests are thought to be formed predominantly by deterministic processes (Chase and Myers, 2011). For these plants, flowering time is driven by the avoidance of harsh environmental conditions. When this occurs, the mid-domain flowering patterns may deviate from null model expectations. At lower latitudes, where environmental conditions are mild, plant assemblages (e.g., tropical and subtropical forests) are formed by stochastic or neutral processes (Hubbell, 2001; Legendre et al., 2009). Under these conditions the null model (i.e. the mid-domain hypothesis) may predict patterns of flowering phenology (Pau et al., 2011; Craine et al., 2012). However, subtropical regions (e.g., South China) can experience large seasonal variations in temperature, with short-lived cold surges that restrict flowering for most species (Dudgeon and Corlett, 1994). Thus, it is important to examine whether the contribution of the mid-domain effect to patterns of phenological diversity varies with latitude.

Flowering in both woody species and herbaceous species is thought to be controlled by a few key environmental cues. Most temperate woody plants flower in response to temperature (Weiser, 1970; Du et al., 2020), although the flowering of woody species, especially in seasonal tropical forests, is often induced by rainfall (Rathcke and Lacey, 1985). The onset of flowering in woody species may also be influenced by other factors such as solar irradiance (van Schaik et al., 1993). Phenological periods of woody species may converge on the same few months with favorable environmental conditions despite the potential costs of competition (Dunn et al., 2007). For many herbs, flowering has been shown to be affected by cold temperature (e.g., Borchert, 1983; Inouye, 2008; Panchen et al., 2012; Matthews and Mazer, 2016; Augspurger and Salk, 2017; Ehrlen and Valdes, 2020). For example, shoot elongation and flowering of herbs can occur very quickly after snow-release or temperature increase (Billings and Mooney, 1968).

In this study, we tested whether peak flowering time is at least partially explained by the mid-domain effect. For this purpose, we used phenological data of 16,267 herbaceous and woody species from the provincial *Flora* in China and species distribution data from the Chinese Vascular Plant Distribution Database to explore the relationships between observed number of species flowering and the number of flowering species as predicted by the mid-domain effect, as well as three climatic variables (mean minimum monthly temperature, mean monthly precipitation, and mean monthly sunshine duration). We hypothesized that (1) the mid-domain effect partially explains variation in peak flowering time of plants at the regional scale; (2) the mid-domain effect is more pronounced in flowering patterns of species from lower latitudes than from those at high latitudes; (3) the flowering patterns of both herbaceous and woody species are correlated with both the mid-domain effect and environmental variables.

## 2 Materials and methods

### 2.1 Species distribution and phenology data

Species distribution data was obtained from the Chinese Vascular Plant Distribution Database (Xu et al., 2018), which was compiled from over six million specimens and more than 1000 published floras, checklists, and inventory reports. All records in this database have been georeferenced to the county level, and there are 30,811 species with distribution data.

We studied the mid-domain effect at the provincial level. Flowering phenology data was available from local *Flora* from 27 provinces; county level phenology data does not exist at present. For each province, we scored a species as being present if it was found in any county in the province. Three pieces of information were compiled: the earliest month and the latest of flowering and the growth form (woody or herbaceous). We calculated the midpoint of the flowering period listed in the *Flora*. Although this estimate of flowering time is somewhat biased because right-skewed flowering curves are common, the midpoint has frequently been used in studies of flowering phenology across communities or floras when precise phenological data is unavailable (e.g., Kochmer and Handel, 1986; Johnson, 1993; Wright and Calderon, 1995; Warren et al., 2011; Du et al., 2020). Species with incomplete flowering information were excluded from the study. There were 16,267 species with flowering data, representing 2218 genera and 251 families (5721 woody and 10,546 herbaceous species).

We did not test the mid-domain effect at the whole-country level. This is because in a country level analysis, a species’ flowering date accounts for flowering dates across all the provinces where the species is distributed (meaning widely-distributed species that flower at different times in high and low latitude provinces will have wide flowering ranges). This issue does not apply to province-level analyses, where province-level flowering data can be appropriately used.

### 2.2 Climatic data

Flowering in plant species has been shown to be regulated by three key environmental variables: temperature, precipitation, and solar irradiance (Frankie et al., 1974; Reich and Borchert, 1984; Wright and van Schaik, 1994). To examine the influence of climate on the number of species flowering each month, we extracted data from 2015 to 2020 on three environmental variables from the China Meteorological Data Service Centre (http://data.cma.cn): mean minimum monthly temperature (°C, T_min_), mean monthly precipitation (mm, MMP), and mean monthly sunshine duration (h, Sunshine). Minimum temperature, rather than mean temperature, is usually thought to determine the species distribution and flowering phenology (Augspurger and Salk, 2017; Ehrlen and Valdes, 2020). Sunshine acts as an index of irradiance and is defined as the sum of all time periods during the day when the direct solar irradiance equals or exceeds 120 W/m^2^.

### 2.3 Data analyses

The mid-domain effect null model was used to test the influence of geometric constraints (in the complete absence of any supposition of environmental gradients within the domain; for details, see Colwell and Lees, 2000) on the temporal patterns of species richness along a monthly gradient. In addition, the null model was constrained by the empirical ranges of the flowering periods in our study. We explored whether the null model of a phenological mid-domain effect could qualitatively match the observed mid-season increase in species flowering. We first tested the mid-domain effect for each of the 27 provinces. We used the random placement model (model 4 in Colwell's RangeModel; see Colwell and Lees, 2000) to generate a predicted species richness pattern under geometric constraints. A flowering season (the domain) of *n* months was considered. To use a mid-domain model to investigate the temporal patterns of flowering species richness, we reduced the occurrence of each species' observed flowering period to two parameters—its midpoint and its duration. The calendar year was divided into 12 month-long segments. We used different flowering domains for different provinces. For example, in Heilongjiang Province, we set April as the start season and October as the end season, according to the flowering duration of all species from this province. To apply mid-domain models to month segments, observed flowering ranges of species were randomly assigned to the domain. This model randomly chose ranges from the empirical frequency distributions of range sizes and placed them within the domain at random. This practice thus eliminated bias due to the difference between theoretical frequency distributions of range sizes and empirical ones. We then “reshuffled” the observed ranges in the domain by resampling with replacement so that each range was assigned a new random midpoint based on possible midpoints imposed by the range of the flowering period (Morales et al., 2005). We implemented the simulation program in RangeModel v.5 (Colwell, 2004). We conducted 5000 simulations of random range placement within the bounded domain. The computed mean richness and its 95% confidence interval was used to evaluate the explanatory power of the mid-domain effect for the empirical number of species flowering each month.

We first conducted generalized linear models to evaluate the relationships between the observed number of species flowering and the mid-domain effect model-predicted number of species flowering. The R^2^ and *P* values from linear regressions of the observed number of species flowering against the predicted number of species flowering were computed to assess the fit of the mid-domain effect models. We also conducted linear regressions between variance explained by the mid-domain effect models as a function of latitude for all pooled species, for herbs and woody species, respectively.

Because the shape and midpoint of a modeled mid-domain effect are dependent on boundary locations, caution should be taken when fitting a model for mid-domain effect if flowering is possible all year long, as in the southernmost provinces. Here we tested the sensitivity of our conclusions to a rule-defined method of setting the boundaries. We did this by computing the dates on which 2.5% and 97.5% of cumulative flowering records had occurred in each province and used the dates as domain boundaries. Then, we repeated the computations for 5% and 95% of cumulative flowering records instead to test the sensitivity of the patterns to this method. We included the results of the 5% and 95% cumulative flowering records in the main text because the boundaries were stricter, but the results of the 2.5% and 97.5% cumulative flowering records can be found in the supplementary materials.

To examine the relative importance of the mid-domain effect model and climatic variables in shaping the phenological patterns for pooled species, for herbaceous species and for woody species, linear mixed-effects models were used to model the number of species flowering (log-transformed) as a function of mid-domain effect (MDE, log-transformed), mean minimum monthly temperature (°C, T_min_), mean monthly precipitation (mm, MMP), and mean monthly sunshine duration (h, Sunshine), with a random intercept for province. The ‘*lmer*’ function in R package ‘*lme4*’ was used to create the model. We used the variance inflation factor (VIF) to assess the multicollinearity among predictors, and we found that all VIFs were less than 3, indicating that collinearity was not a problem for our multiple regression models. The individual contribution of each selected fixed-factor was determined using the ‘*glmm.hp*’ package in R, which was developed to evaluate the relative importance of predictors in linear mixed models (Lai et al., 2022, 2023). All calculations were performed using the software R v.4.3.1 (R Core Team, 2023).

## 3 Results

### 3.1 Mid-domain effect in peak flowering phenology

**Fig. 1:**
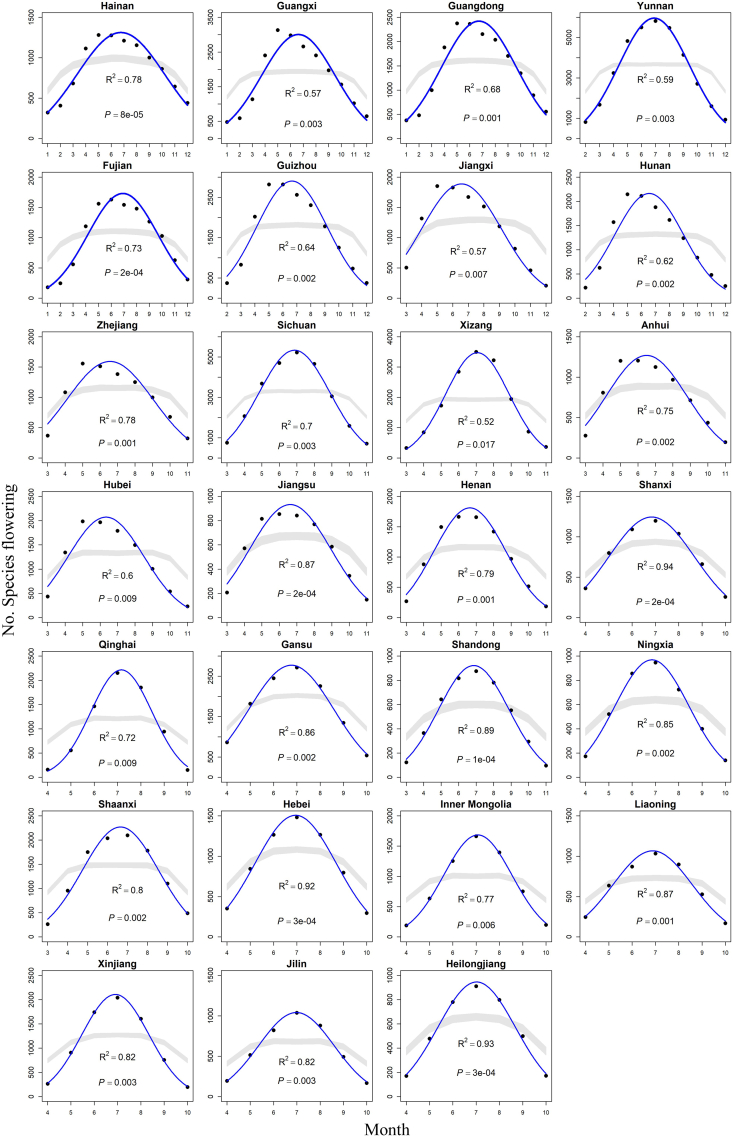
Observed and null model-predicted flowering diversity for 27 provinces in China, based on dates for which 5% and 95% of cumulative flowering records occurred. The fitted values (solid lines) and the 95% confidence interval predicted by the mid-domain models (gray bands) are shown in the plots. The R^2^ and *P*-values reflect the relationship between the observed data and the predicted values from the mid-domain model.

**Fig. 2:**
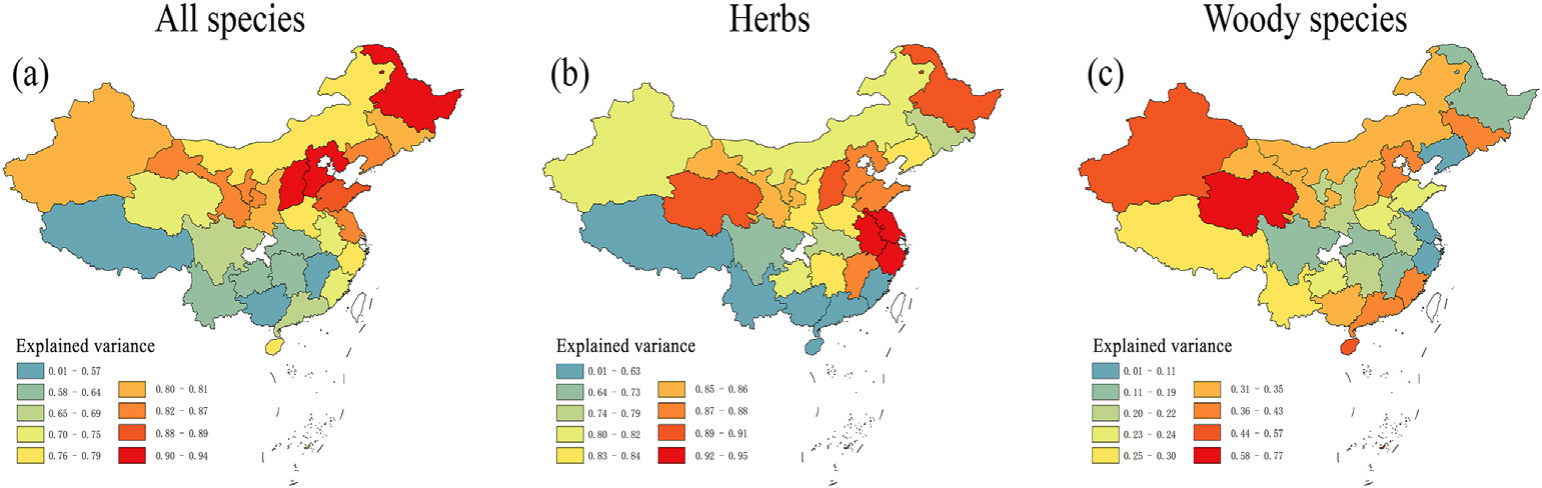
The geographic patterns of the variance explained by the mid-domain effect models in China estimated at the provincial level for (a) all species pooled, (b) herbs and (c) woody species based on dates for which 5% and 95% of cumulative flowering records occurred.

For the dates on which 5% and 95% of cumulative flowering records occurred, the null model explained a significant proportion of the variation in the number of flowering species across the temporal gradient in all 27 provinces for all species pooled (Fig. 1, Fig. 2a) and for herbaceous species (Table 1 and Fig. 2b). For woody species, we found a significant or marginally significant (0.05 < *P* < 0.10) relationship between the empirical species richness and the predicted richness for 14 out of 27 provinces (Table 1 and Fig. 2c). The mid-domain effect model explained between 51.9% and 94.0% of the variance in the number of flowering species for all species pooled, between 58.7% and 94.5% for herbaceous species, and between 3.5% and 76.7% for woody species (Table 1).

**Table 1:** Summary of the generalized linear models of the relationship between the observed number of species flowering and the mid-domain effect model-predicted number of species flowering, based on dates for which 5% and 95% of cumulative flowering records occurred.

Province	Latitude	All species				Herbaceous species				Woody species			
		F	R^2^	*P*	Period	F	R^2^	*P*	Period	F	R^2^	*P*	Period
Hainan	19.222	40.7	0.783	<0.001	1–12	19.4	0.625	<0.001	1–12	15.8	0.574	0.003	1–12
Guangxi	23.015	15.8	0.573	0.003	1–12	16.6	0.587	0.002	1–12	6.6	0.337	0.028	1–12
Guangdong	23.277	24.8	0.684	0.001	1–12	20.1	0.63	0.001	1–12	7.6	0.376	0.02	1–12
Yunnan	24.141	15.6	0.593	0.003	2–12	14.8	0.605	0.005	3–12	5.5	0.29	0.040	1–12
Fujian	26.004	31.1	0.732	<0.001	1–12	18.5	0.614	0.002	1–12	9.2	0.426	0.013	1–12
Guizhou	26.668	18.9	0.642	0.002	2–12	42.3	0.821	<0.001	3–12	4.4	0.238	0.061	1–12
Jiangxi	27.735	12.9	0.569	0.007	3–12	60.7	0.869	<0.001	3–12	3.3	0.186	0.103	2–12
Hunan	28.016	17.6	0.624	0.002	2–12	48.7	0.841	<0.001	3–12	3.7	0.209	0.088	2–12
Zhejiang	29.105	30.1	0.784	0.001	3–11	132.4	0.943	<0.001	3–11	2.0	0.113	0.198	3–11
Sichuan	30.277	19.6	0.699	0.003	3–11	19.9	0.73	0.004	4–11	2.9	0.196	0.129	3–11
Xizang	31.101	9.6	0.519	0.017	3–11	12.2	0.616	0.013	4–11	4.5	0.306	0.071	3–11
Anhui	32.014	25.3	0.751	0.002	3–11	113.9	0.934	<0.001	3–11	2.9	0.212	0.14	3–10
Hubei	32.014	12.9	0.599	0.009	3–11	31.8	0.794	0.0008	3–11	2.6	0.185	0.159	3–10
Jiangsu	32.472	53.4	0.868	<0.001	3–11	139.3	0.945	<0.001	3–11	2.0	0.109	0.202	3–11
Henan	33.800	31.8	0.794	<0.001	3–11	44.2	0.844	<0.001	3–11	3.3	0.244	0.121	3–10
Shanxi	34.115	95.0	0.94	<0.001	4–10	69.6	0.919	<0.001	4–10	4.9	0.357	0.069	3–10
Qinghai	35.723	16.3	0.719	0.009	4–10	51.2	0.909	0.002	5–10	17.4	0.767	0.014	4–9
Gansu	35.949	37.2	0.858	0.002	4–10	38.3	0.861	0.002	4–10	4.6	0.336	0.077	3–10
Shandong	36.178	69.7	0.896	<0.001	3–11	55.1	0.871	<0.001	3–11	3.3	0.248	0.119	3–10
Ningxia	37.366	35.9	0.853	0.002	4–10	37.3	0.858	0.002	4–10	2.7	0.216	0.164	4–10
Shaanxi	37.699	29.6	0.803	0.002	3–10	34.7	0.849	0.002	4–10	3.1	0.227	0.131	3–10
Hebei	38.222	74.3	0.924	<0.001	4–10	43.6	0.877	0.001	4–10	6.5	0.436	0.044	3–10
Inner Mongolia	41.386	20.9	0.768	0.006	4–10	25.1	0.828	0.007	5–10	4.0	0.332	0.103	4–10
Liaoning	41.474	42.3	0.874	0.001	4–10	31.6	0.836	0.002	4–10	1.2	0.035	0.338	4–9
Xinjiang	42.002	28.2	0.819	0.003	4–10	27.7	0.816	0.003	4–10	8.3	0.548	0.035	4–10
Jilin	43.501	27.8	0.817	0.003	4–10	22.4	0.781	0.005	4–10	4.6	0.373	0.086	3–9
Heilongjiang	46.770	85.2	0.934	<0.001	4–10	54.9	0.9	<0.001	4–10	1.0	0.166	0.365	4–10

Analyses of 2.5% domain and 5% domain generated similar results (Appendix A). This suggests that our results were robust to this sampling strategy.

### 3.2 Latitudinal patterns of mid-domain effect

**Fig. 3:**
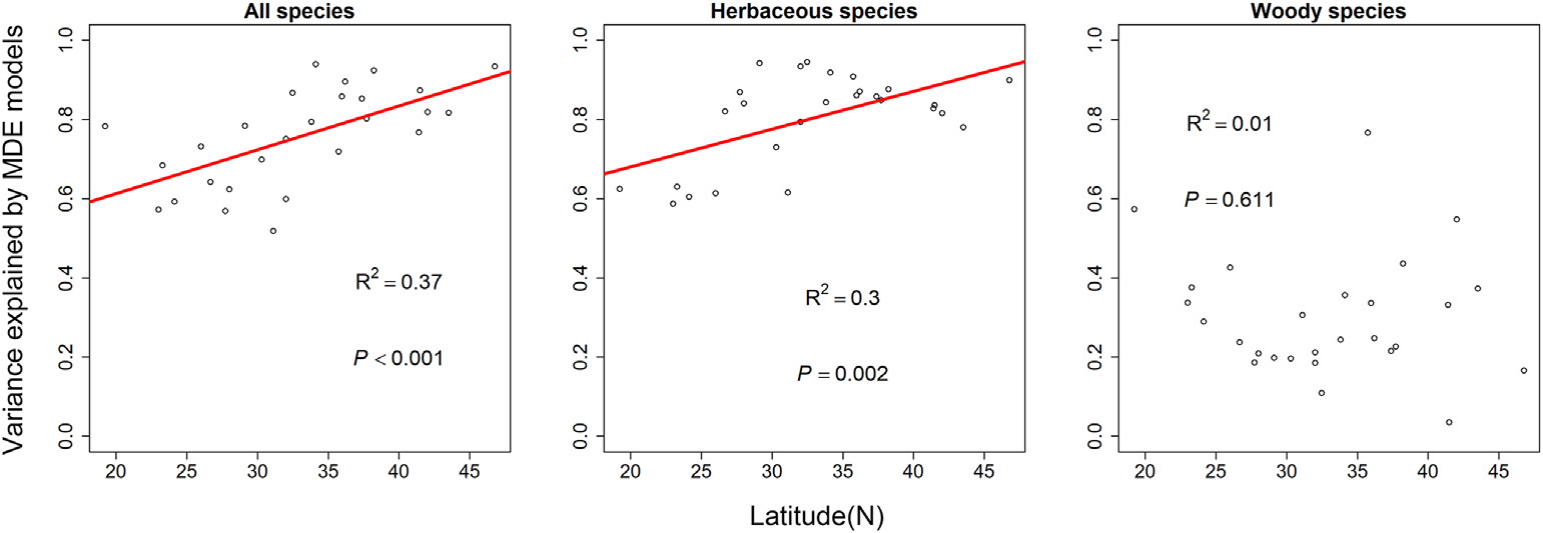
The relationship between the variance explained by the mid-domain effect models and latitude for all species (left panel), herbaceous species (middle panel) and woody species (right panel), based on dates for which 5% and 95% of cumulative flowering records occurred. Each circle represents a single province. The red line is the fitted line from the linear model.

The 5% domain analyses showed that the variance explained by the mid-domain effect models was positively related with latitude for all species pooled (R^2^ = 0.37, *P* < 0.001) and for herbaceous species (R^2^ = 0.30, *P* = 0.002; Fig. 2, Fig. 3a and b). No significant regression was found between variance explained by the mid-domain effect models and latitude for woody species (Fig. 3c). The 2.5% domain analyses generated similar results (Appendix A).

### 3.3 Relationship between climatic variables and the flowering diversity

The observed numbers of species flowering were significantly related to the mid-domain effect model-predicted species richness and to all three climatic variables, but the relationships varied between herbs and woody species (Table 2).

**Table 2:** Parameters from linear mixed-effects models (lmer) modeling the number of species flowering (log-transformed) as a function of mid-domain effect (MDE), mean minimum monthly temperature (°C, T_min_), mean monthly precipitation (mm, MMP), and mean monthly sunshine duration (h, Sunshine), with a random intercept for province, based on dates for which 5% and 95% of cumulative flowering records occurred. ‘I.perc’ column is the individual contribution percentage in the glmm.hp function.

	All species			Herbaceous species			Woody species		
	t	*p*	I.perc (%)	t	*p*	I.perc (%)	t	*p*	I.perc (%)
log(MDE)	14.9	<0.001	49.1	15.7	<0.001	50.8	21.6	<0.001	78.7
Tmin	9.0	<0.001	33.0	15.1	<0.001	33.5	1.4	0.177	9.2
MMP	2.3	0.025	13.5	−0.8	0.422	12.5	3.1	0.002	6.0
Sunshine	2.9	0.004	4.4	−0.6	0.543	3.2	6.9	<0.001	6.1

For herbaceous species, the mid-domain effect had the greatest predictive power in explaining observed numbers of species flowering (50.8% of variance). Mean minimum monthly temperature and mean monthly precipitation were the second and third most important predictors, accounting for 33.5% and 12.5% of the variance, respectively. For woody species, the mid-domain effect had strong predictive power, explaining 78.7% of variance in the observed number of flowering species. Mean minimum monthly temperature accounted for 9.2% of the variance. Mean monthly precipitation and monthly sunshine duration were less informative, explaining less than 7% of variance. Similar results were found when we repeated the analyses using the 2.5% domain data (Appendix A).

## 4 Discussion

### 4.1 Mid-domain effect in peak flowering phenology

We found that interspecific overlap in the temporal ranges of flowering phenology within a defined temporal domain resulted in a mid-domain peak. The prevalent mid-domain ‘saddle’ in the panels of Fig. 1 is likely a consequence of many small and relatively equal-sized ‘flowering periods’ (Colwell et al., 2009). Our results suggest that the null model of a phenological mid-domain effect explains a large percentage of the temporal variation in flowering diversity. Thus, future studies should use mid-domain null models to examine factors that drive seasonal patterns of flowering diversity.

This mid-domain effect is consistent with a previous study that examined flowering phenology (Morales et al., 2005), although our study has some new findings. Firstly, while Morales et al. (2005) included only dozens of species, our study on mid-domain effect in flowering phenology included more than 16,000 species. That is, our study suggests that phenological mid-domain effects may be a general phenomenon. Secondly, Morales et al. (2005) only included forbs, whereas we found that the mid-domain effect may explain the flowering patterns of both herbs and woody species. Thirdly, Morales et al. (2005) study was conducted in sub-alpine communities in a temperate region, whereas our study covered temperate, subtropical, and north tropical regions. In short, our findings indicate that the mid-domain effect can explain patterns of flowering diversity in various ecosystems.

Perhaps the most novel finding of our study is that the mid-domain effect model explained a greater proportion of the variance in flowering patterns at higher latitudes than at lower latitudes, which is not what we predicted. However, this latitudinal pattern was only observed for herbaceous species. In other words, in regions with distinct flowering seasons, more species tend to flower near the temporal boundaries of the flowering season than near the middle (Colwell and Lees, 2000). Our results suggest that geometric constraints should be largely considered as drivers of patterns of flowering diversity in temperate ecosystems. Consequently, previous suggestions that plant assemblages are dominated by neutral processes at lower latitudes (like tropical rainforests) and by deterministic processes at higher latitudes (Hubbell, 2001; Rangel and Diniz-Filho, 2005; Clark, 2009) may be only suitable for spatial niches and not for temporal niches. Our findings suggest that plant flowering phenology in lower latitudes may be more influenced by species interactions and by environmental conditions.

Another potential explanation for the better fit of the mid-domain model at high latitudes is that the shorter growing season found at higher latitudes means that the effective domain (in this case, the number of months over which species might flower) is smaller at higher latitudes, which may have resulted in a more strongly peaked mid-domain effect (Colwell and Lees, 2000) and potentially caused a greater match between the predictions of our null model and the observed data.

No latitudinal patterns were found for woody species, possibly because environmental factors, and especially biotic factors, influence flowering phenology strongly at both higher and lower latitudes. This theory is supported by previous findings that flowering patterns in both temperate and tropical forests are driven by climatic variables such as temperature, precipitation and photoperiod (Borchert et al., 2005; Stevenson et al., 2008; Pau et al., 2011; Flynn and Wolkovich, 2018). Compared to herbaceous species, the reproductive phenology of woody species may be more determined by biotic interactions, including both selection to coincide with the seasonality of mutualists (e.g., pollinators and seed dispersers) and selection to avoid the seasonality of pests (e.g., herbivores and seed predators) (Stiles, 1977; Brody, 1997). For example, woody species, and not herbs, exhibit mast flowering and fruiting in both temperate and tropical forests, with synchronous, species-wide, mass productions of flowers and seeds (Appanah, 1993; Wesolowski et al., 2015). The evolutionary mechanisms at work in masting include seed predator satiation (Janzen, 1971) and increased efficiency in pollination or fruit dispersal (Norton and Kelly, 1988; Kelly, 1994). Because seed size is usually small for herbs, herbaceous species are hard-pressed to satiate seed predators. Our study shows that only by considering all possible abiotic and biotic variables that affect the temporal patterns of flowering phenology may we gain a complete understanding of the evolution of flowering phenology.

### 4.2 Relationship between climatic variables and flowering diversity

To our knowledge, our study is the first to put climatic variables and mid-domain effect into one model to assess the relative importance of mid-domain effect and climatic factors in driving plant phenology patterns. We found that flowering diversity of herbaceous plant assemblages is most strongly related with the mid-domain effect, mean minimum monthly temperature, and mean monthly precipitation. This suggests that herbaceous plants may begin to flower earlier as the climate warms. This is supported by the finding that the phenology of herbs is driven by minimum temperature (e.g., Inouye, 2008; Matthews and Mazer, 2016; Augspurger and Salk, 2017). The flowering diversity of woody plant assemblages is primarily related to the mid-domain effect and temperature. In contrast to herbaceous plants, flower development in many trees is not continuous from flower induction to anthesis but may become temporarily arrested at some intermediate stage (Borchert, 1983). Final development of flower buds and anthesis of woody species occurs many months after flower initiation, and during this period, flowering may need other stimuli such as warm temperatures (van Schaik et al., 1993). One of the main findings of our study is that the mid-domain effect is the most important variable for explaining the phenological patterns of both herbaceous and woody species.

Our finding that the flowering diversities of woody and herbaceous plant assemblages are determined by different drivers suggests that any climate-driven and/or random reassembly will likely follow distinct trajectories (Ackerly, 2003). This might have major consequences for community reshuffling under climate change. We argue that the mid-domain effect must be considered, in addition to abiotic and biotic factors, when assessing the patterns of phenology in the future. Specifically, we should at least combine the mid-domain effect null model and environmental variables into a single model to investigate the relative importance of each variable on the number of species flowering each period. We believe that future studies should explore whether warming temperature is more important than the mid-domain effect under climate change scenarios and whether the relative importance of the mid-domain effect in explaining flowering diversity changes for herbaceous versus woody species in temperate, subtropical, and tropical forests.

### 4.3 Limitations of the study

Three aspects of our study require caution when interpreting the results. Firstly, assessing the contribution of the mid-domain effect to empirical patterns requires careful placement of domain boundaries because the shape and midpoint of a modeled the mid-domain effect are highly dependent on boundary locations. This problem is intrinsically more challenging for annual phenological datasets, which are cyclic rather than intrinsically bounded, unlike spatial domains. In this study, we tested the sensitivity of our conclusions to a rule-defined method of setting the boundaries by computing domain boundaries for each province based on the dates on which 2.5% and 97.5% of cumulative flowering records have occurred. Then, we tried boundaries based on 5% and 95% instead to test the sensitivity of the patterns to this method. We found that both analyses generated similar results, even as the domains changed, suggesting that our results were robust using this sampling strategy.

Secondly, compared to research by Morales et al. (2005), our study had larger time increments because we used monthly phenology data. It would have been more accurate to use more precise phenology data for each species in each province, but this data does not exist at present. We suggest that future studies use observer-based phenology data to test the effects of the mid-domain effect on flowering diversity along a latitudinal gradient.

In addition, we should be careful about the collinearity of the mid-domain effect with environmental variables (Letten et al., 2013). The mid-domain effects expected to be related to both abiotic parameters and biotic components, such as species interactions, which means that a species range is dependent on both the environment and other species. Therefore, caution is required when interpreting our results, although many previous studies also incorporated the mid-domain effect and environmental variables into multiple linear regression models to examine which variable contributed the most to explaining species richness gradients (e.g., ChangBae et al., 2013; Feng et al., 2017; Sun et al., 2020). In our study, we calculated the variance inflation factor to assess the multicollinearity among predictors, and we found that VIF was low, which suggests that collinearity was not a major problem. We also used the newly developed ‘*glmm.hp*’ R package to quantify the importance of each predictor variable, therefore facilitating our interpretation of the importance of each predictor, particularly when these variables were correlated (Lai et al., 2022). Future work should improve methodological approaches to account for the collinearity of the mid-domain effect with environmental variables.

## 5 Conclusions

Using the largest flowering phenology dataset to date, we provide independent confirmation that mid-domain models qualitatively matched patterns of flowering species richness. The mid-domain effect was among the most important variables in explaining the flowering patterns for both herbaceous and woody species, but it has been neglected in previous studies. Identifying the relative importance of mid-domain effects and abiotic and biotic processes on phenological patterns presents an interesting challenge that deserves further attention. Our results suggest that geometric constraints are of sufficient magnitude to warrant further consideration as drivers of flowering seasonality for temperate, subtropical and tropical forests. Our findings also indicate that other phenological patterns, such as leaf flush and fruit ripening times, might display mid-domain effects as well. These possibilities would be worthwhile directions to pursue in future research.

## Data availability

The data that supports the findings of this study is openly available through Github: https://github.com/YanjunDu/MDE.

## CRediT authorship contribution statement

**Yanjun Du:** Writing – original draft, Investigation, Funding acquisition, Formal analysis, Conceptualization. **Rongchen Zhang:** Writing – original draft, Data curation. **Xinran Tang:** Writing – original draft, Formal analysis, Data curation. **Xinyang Wang:** Writing – original draft, Formal analysis, Data curation. **Lingfeng Mao:** Formal analysis, Data curation, Conceptualization. **Guoke Chen:** Formal analysis, Data curation, Conceptualization. **Jiangshan Lai:** Methodology, Formal analysis, Data curation, Conceptualization. **Keping Ma:** Writing – original draft, Supervision, Investigation, Conceptualization.

## Declaration of competing interest

The authors declare that they have no known competing financial interests or personal relationships that could have appeared to influence the work reported in this paper.

## References

[bib1] Ackerly D.D. (2003). Community assembly, niche conservatism, and adaptive evolution in changing environments. Int. J. Plant Sci..

[bib2] Appanah S. (1993). Mass flowering of dipterocarp forests in the aseasonal tropics. J. Biosci..

[bib3] Augspurger C.K., Salk C.F. (2017). Constraints of cold and shade on the phenology of spring ephemeral herb species. J. Ecol..

[bib4] Billings W.D., Mooney H.A. (1968). The ecology of arctic and alpine plants. Biol. Rev..

[bib5] Borchert R. (1983). Phenology and control of flowering in tropical trees. Biotropica.

[bib6] Borchert R., Robertson K., Schwartz M.D. (2005). Phenology of temperate trees in tropical climates. Int. J. Biometeorol..

[bib7] Brody A.K. (1997). Effects of pollinators, herbivores, and seed predators on flowering phenology. Ecology.

[bib8] CaraDonna P.J., Iler A.M., Inouye D.W. (2014). Shifts in flowering phenology reshape a subalpine plant community. Proc. Natl. Acad. Sci. U.S.A..

[bib9] ChangBae L., Chun J.H., Cho H.J. (2013). Elevational patterns and determinants of plant diversity in the Baekdudaegan Mountains, South Korea: species vs. functional diversity. Chin. Sci. Bull..

[bib10] Chase J.M., Myers J.A. (2011). Disentangling the importance of ecological niches from stochastic processes across scales. Philos. Trans. R. Soc. B-Biol. Sci..

[bib11] Chen Z., Li X., Song W. (2020). Small mammal species richness and turnover along elevational gradient in Yulong Mountain, Yunnan, Southwest China. Ecol. Evol..

[bib12] Chettri B., Acharya B.K. (2020). Distribution of amphibians along an elevation gradient in the Eastern Himalaya, India. Basic Appl. Ecol..

[bib13] Clark J.S. (2009). Beyond neutral science. Trends Ecol. Evol..

[bib14] Cleland E.E., Chiariello N.R., Loarie S.R. (2006). Diverse responses of phenology to global changes in a grassland ecosystem. Proc. Natl. Acad. Sci. U.S.A..

[bib15] Colwell R.K. (2004). RangeModel: a Monte Carlo simulation tool for assessing geometric constraints on species richness, version 4b8. User’s guide and application.

[bib16] Colwell R.K., Gotelli N.J., Rahbek C. (2009). Peaks, plateaus, canyons, and craters: the complex geometry of simple mid-domain effect models. Evol. Ecol. Res..

[bib17] Colwell R.K., Hurtt G.C. (1994). Nonbiological gradients in species richness and a spurious Rapoport effect. Am. Nat..

[bib18] Colwell R.K., Lees D.C. (2000). The mid-domain effect: geometric constraints on the geography of species richness. Trends Ecol. Evol..

[bib19] Colwell R.K., Rahbek C., Gotelli N.J. (2004). The mid-domain effect and species richness patterns: what have we learned so far?. Am. Nat..

[bib20] Craine J.M., Wolkovich E.M., Towne E.G. (2012). The roles of shifting and filtering in generating community-level flowering phenology. Ecography.

[bib21] Du Y.J., Mao L.F., Queenborough S.A. (2020). Macro-scale variation and environmental predictors of flowering and fruiting phenology in the Chinese angiosperm flora. J. Biogeogr..

[bib22] Dudgeon D., Corlett R.T. (1994).

[bib23] Dunn R.R., Parker C.R., Sanders N.J. (2007). Temporal patterns of diversity: assessing the biotic and abiotic controls on ant assemblages. Biol. J. Linn. Soc..

[bib24] Ehrlen J., Valdes A. (2020). Climate drives among-year variation in natural selection on flowering time. Ecol. Lett..

[bib25] Feng C., Wu Y., Tian F. (2017). Elevational diversity gradients of Tibetan loaches: the relative roles of ecological and evolutionary processes. Ecol. Evol..

[bib26] Fenner M. (1998). The phenology of growth and reproduction in plants. Perspect. Plant Ecol..

[bib27] Flynn D.F.B., Wolkovich E.M. (2018). Temperature and photoperiod drive spring phenology across all species in a temperate forest community. New Phytol..

[bib28] Frankie G.W., Baker H.G., Opler P.A. (1974). Comparative phenological studies of trees in tropical wet and dry forests in the lowlands of Costa Rica. J. Ecol..

[bib29] Heithaus E.R. (1974). The role of plant-pollinator interactions in determining community structure. Ann. Mo. Bot. Gard..

[bib30] Hubbell S.P. (2001).

[bib31] Inouye D.W. (2008). Effects of climate change on phenology, frost damage, and floral abundance of montane wildflowers. Ecology.

[bib32] Janzen D.H. (1971). Seed predation by animals. Annu. Rev. Ecol. Syst..

[bib33] Johnson S.D. (1993). Climatic and phylogenetic determinants of flowering seasonality in the Cape flora. J. Ecol..

[bib34] Kelly D. (1994). The evolutionary ecology of mast seeding. Trends Ecol. Evol..

[bib35] Kochmer J.P., Handel S.N. (1986). Constraints and competition in the evolution of flowering phenology. Ecol. Monogr..

[bib36] Lai J., Zou Y., Zhang S. (2022). glmm.hp: an R package for computing individual effect of predictors in generalized linear mixed models. J. Plant Ecol..

[bib37] Lai J., Zhu W., Cui D. (2023). Extension of the glmm.hp package to zero-inflated generalized linear mixed models and multiple regression. J. Plant Ecol..

[bib38] Legendre P., Mi X., Ren H. (2009). Partitioning beta diversity in a subtropical broad-leaved forest of China. Ecology.

[bib39] Letten A.D., Lyons S.K., Moles A.T. (2013). The mid-domain effect: it's not just about space. J. Biogeogr..

[bib40] Matthews E.R., Mazer S.J. (2016). Historical changes in flowering phenology are governed by temperature×precipitation interactions in a widespread perennial herb in western North America. New Phytol..

[bib41] Morales M.A., Dodge G.J., Inouye D.W. (2005). A phenological mid-domain effect in flowering diversity. Oecologia.

[bib42] Morin X., Chuine I. (2006). Niche breadth, competitive strength and range size of tree species: a trade-off based framework to understand species distribution. Ecol. Lett..

[bib43] Norton D.A., Kelly D. (1988). Mast seeding over 33 years by *Dacrydium cupressinum* Lamb. (rimu) (Podocarpaceae) in New Zealand: the importance of economies of scale. Funct. Ecol..

[bib44] Panchen Z.A., Primack R.B., Aniśko T. (2012). Herbarium specimens, photographs, and field observations show Philadelphia area plants are responding to climate change. Am. J. Bot..

[bib45] Pau S., Wolkovich E.M., Cook B.I. (2011). Predicting phenology by integrating ecology, evolution and climate science. Global Change Biol..

[bib46] Post E., Forchhammer M.C., Stenseth N.C. (2001). The timing of life-history events in changing climate. Proc. Roy. Soc. B-Biol. Sci..

[bib47] R Development Core Team (2023). http://www.Rproject.org.

[bib48] Rangel T.F., Diniz-Filho J.A. (2005). Neutral community dynamics, the mid-domain effect and spatial patterns in species richness. Ecol. Lett..

[bib49] Rathcke B., Lacey E.P. (1985). Phenological patterns of terrestrial plants. Annu. Rev. Ecol. Evol. Syst..

[bib50] Reich P.B., Borchert R. (1984). Water stress and tree phenology in a tropical dry forest in the lowlands of Costa Rica. J. Ecol..

[bib51] Siqueira C.C., Vrcibradic D., Almeida-Gomes M. (2021). Assessing the importance of reproductive modes for the evaluation of altitudinal distribution patterns in tropical frogs. Biotropica.

[bib52] Stevenson P.R., Castellanos M.C., Cortés A.I. (2008). Flowering patterns in a seasonal tropical lowland forest in western Amazonia. Biotropica.

[bib53] Stiles F.G. (1977). Coadapted competitors: the flowering seasons of hummingbird-pollinated plants in a tropical forest. Science.

[bib54] Sun L., Luo J., Qian L. (2020). The relationship between elevation and seed-plant species richness in the Mt. Namjagbarwa region (Eastern Himalayas) and its underlying determinants. Glob. Ecol. Conserv..

[bib55] Thies W., Kalko E.K.V. (2004). Phenology of neotropical pepper plants (Piperaceae) and their association with their main dispersers, two short-tailed fruit bats, *Carollia perspicillata* and *C. castanea* (Phyllostomidae). Oikos.

[bib56] van Schaik C.P., Terborgh J.W., Wright S.J. (1993). The phenology of tropical forests: adaptive significance and consequences for primary consumers. Annu. Rev. Ecol. Evol. Syst..

[bib57] Warren B.H., Bakker F.T., Bellstedt D.U. (2011). Consistent phenological shifts in the making of a biodiversity hotspot: the Cape flora. BMC Evol. Biol..

[bib58] Weiser C.J. (1970). Cold resistance and injury in woody plants: knowledge of hardy plant adaptations to freezing stress may help us to reduce winter damage. Science.

[bib59] Wesolowski T., Rowinski P., Maziarz M. (2015). Interannual variation in tree seed production in a primeval temperate forest: does masting prevail?. Eur. J. For. Res..

[bib60] Wright S.J. (1996). Tropical Forest Plant Ecophysiology.

[bib61] Wright S.J., Calderon O. (1995). Phylogenetic patterns among tropical flowering phenologies. J. Ecol..

[bib62] Wright S.J., van Schaik C.P. (1994). Light and the phenology of tropical trees. Am. Nat..

[bib63] Xu W.B., Svenning J.C., Chen G.K. (2018). Plant geographical range size and climate stability in China: growth form matters. Global Ecol. Biogeogr..

