# Supplementary material for: The mid-domain effect in flowering phenology
Source: Plant Divers. 2024 May 25;46(4):502–9. doi: 10.1016/j.pld.2024.05.005 (PMC11390702; doi:10.1016/j.pld.2024.05.005)

**Supplementary Data for**

**The mid-domain effect in flowering phenology**

Yanjun Du, Rongchen Zhang, Xinran Tang, Xinyang Wang, Lingfeng Mao, Guoke Chen, Jiangshan Lai, Keping Ma

Table S1. Summary of the generalized linear models of the relationship between the observed number of species flowering and the mid-domain effect model-predicted number of species flowering, based on the dates on which 2.5% and 97.5% of cumulative flowering records had occurred.

|  |  | All species | | | |  | Herbaceous species | | | |  | Woody species | | | |
| --- | --- | --- | --- | --- | --- | --- | --- | --- | --- | --- | --- | --- | --- | --- | --- |
| Province | Latitude | F | R^2^ | P | Period |  | F | R^2^ | P | Period |  | F | R^2^ | P | Period |
| Hainan | 19.222 | 40.7 | 0.783 | <0.001 | 1-12 |  | 19.4 | 0.625 | 0.00134 | 1-12 |  | 15.8 | 0.574 | 0.003 | 1-12 |
| Guangxi | 23.015 | 15.8 | 0.573 | 0.003 | 1-12 |  | 16.6 | 0.587 | 0.002 | 1-12 |  | 6.6 | 0.337 | 0.0281 | 1-12 |
| Guangdong | 23.277 | 24.8 | 0.684 | 0.001 | 1-12 |  | 20.1 | 0.634 | 0.0011 | 1-12 |  | 7.6 | 0.376 | 0.0201 | 1-12 |
| Yunnan | 24.141 | 9.7 | 0.44 | 0.011 | 1-12 |  | 6.1 | 0.32 | 0.0323 | 1-12 |  | 5.5 | 0.29 | 0.0404 | 1-12 |
| Fujian | 26.004 | 31.1 | 0.732 | 0.0002 | 1-12 |  | 18.5 | 0.614 | 0.002 | 1-12 |  | 9.2 | 0.426 | 0.013 | 1-12 |
| Guizhou | 26.668 | 12.3 | 0.507 | 0.006 | 1-12 |  | 12.0 | 0.5 | 0.0061 | 1-12 |  | 4.4 | 0.238 | 0.061 | 1-12 |
| Jiangxi | 27.735 | 12.3 | 0.507 | 0.006 | 1-12 |  | 14.5 | 0.55 | 0.00346 | 1-12 |  | 2.5 | 0.118 | 0.147 | 1-12 |
| Hunan | 28.016 | 11.8 | 0.496 | 0.006 | 1-12 |  | 14.9 | 0.56 | 0.00314 | 1-12 |  | 3.1 | 0.16 | 0.107 | 1-12 |
| Zhejiang | 29.105 | 16.3 | 0.605 | 0.003 | 2-12 |  | 43.2 | 0.824 | 0.0002 | 3-12 |  | 2.3 | 0.113 | 0.166 | 2-12 |
| Sichuan | 30.277 | 7.6 | 0.399 | 0.022 | 2-12 |  | 13.4 | 0.608 | 0.008 | 3-11 |  | 2.7 | 0.135 | 0.131 | 1-12 |
| Xizang | 31.101 | 5.5 | 0.333 | 0.047 | 3-12 |  | 12.2 | 0.616 | 0.013 | 4-11 |  | 3.7 | 0.215 | 0.085 | 1-11 |
| Anhui | 32.014 | 16.1 | 0.602 | 0.003 | 2-12 |  | 24.5 | 0.702 | 0.0008 | 2-12 |  | 2.0 | 0.108 | 0.203 | 3-11 |
| Hubei | 32.014 | 7.9 | 0.411 | 0.019 | 2-12 |  | 31.8 | 0.794 | 0.0008 | 3-11 |  | 1.9 | 0.086 | 0.197 | 2-12 |
| Jiangsu | 32.472 | 23.7 | 0.694 | <0.001 | 2-12 |  | 26.5 | 0.718 | 0.0006 | 2-12 |  | 3.9 | 0.223 | 0.081 | 2-12 |
| Henan | 33.800 | 31.8 | 0.794 | <0.001 | 3-11 |  | 44.2 | 0.844 | 0.0003 | 3-11 |  | 3.0 | 0.183 | 0.121 | 2-11 |
| Shanxi | 34.115 | 23.7 | 0.739 | 0.002 | 3-11 |  | 26.8 | 0.786 | 0.002 | 4-11 |  | 4.9 | 0.357 | 0.069 | 3-10 |
| Qinghai | 35.723 | 16.3 | 0.719 | 0.009 | 4-10 |  | 13.0 | 0.667 | 0.015 | 4-10 |  | 5.8 | 0.443 | 0.061 | 4-10 |
| Gansu | 35.949 | 13.3 | 0.607 | 0.008 | 3-11 |  | 11.4 | 0.565 | 0.012 | 3-11 |  | 3.2 | 0.128 | 0.115 | 3-11 |
| Shandong | 36.178 | 69.7 | 0.896 | <0.001 | 3-11 |  | 55.1 | 0.871 | 0.0001 | 3-11 |  | 5.3 | 0.349 | 0.055 | 2-10 |
| Ningxia | 37.366 | 35.9 | 0.853 | 0.002 | 4-10 |  | 37.3 | 0.858 | 0.002 | 4-10 |  | 5.4 | 0.384 | 0.059 | 3-10 |
| Shaanxi | 37.699 | 13.8 | 0.616 | 0.007 | 3-11 |  | 13.0 | 0.599 | 0.009 | 3-11 |  | 1.3 | 0.126 | 0.169 | 2-11 |
| Hebei | 38.222 | 15.1 | 0.638 | 0.006 | 3-11 |  | 18.5 | 0.714 | 0.005 | 4-11` |  | 6.5 | 0.436 | 0.044 | 3-10 |
| Inner Mongolia | 41.386 | 20.9 | 0.768 | 0.006 | 4-10 |  | 15.7 | 0.71 | 0.011 | 4-10 |  | 6.7 | 0.449 | 0.041 | 3-10 |
| Liaoning | 41.474 | 42.3 | 0.874 | 0.001 | 4-10 |  | 31.6 | 0.836 | 0.002 | 4-10 |  | 3.4 | 0.289 | 0.122 | 3-9 |
| Xinjiang | 42.002 | 28.2 | 0.819 | 0.003 | 4-10 |  | 27.7 | 0.816 | 0.003 | 4-10 |  | 8.3 | 0.548 | 0.035 | 4-10 |
| Jilin | 43.501 | 27.8 | 0.817 | 0.003 | 4-10 |  | 22.4 | 0.781 | 0.005 | 4-10 |  | 3.2 | 0.241 | 0.123 | 3-10 |
| Heilongjiang | 46.770 | 15.9 | 0.68 | 0.007 | 4-11 |  | 18.2 | 0.711 | 0.005 | 4-11` |  | 2.8 | 0.201 | 0.148 | 3-10 |

Table S2. Parameters from linear mixed-effects models (lmer) modeling the number of species flowering (log-transformed) as a function of mid-domain effect (MDE), mean minimum monthly temperature (℃, T_min_), mean monthly precipitation (mm, MMP), and mean monthly sunshine duration (h, Sunshine), with a random intercept for province, based on the dates on which 2.5% and 97.5% of cumulative flowering records had occurred. ‘I.perc’ column is the individual contribution percentage in the glmm.hp() function.

|  | All species | | |  | Herbaceous species | | |  | Woody species | | |
| --- | --- | --- | --- | --- | --- | --- | --- | --- | --- | --- | --- |
|  | t | p | I.perc(%) |  | t | p | I.perc(%) |  | t | p | I.perc(%) |
| log(MDE) | 10.1 | <0.001 | 43.8 |  | 13.0 | <0.001 | 43.4 |  | 19.8 | <0.001 | 64.3 |
| Tmin | 13.3 | <0.001 | 36.2 |  | 18.5 | <0.001 | 37.7 |  | 5.8 | <0.001 | 20.8 |
| MMP | -1.5 | 0.124 | 12.3 |  | -2.7 | 0.008 | 13.3 |  | 1.5 | 0.135 | 8.0 |
| Sunshine | 3.1 | 0.002 | 7.7 |  | -0.5 | 0.611 | 5.6 |  | 7.2 | <0.001 | 6.9 |


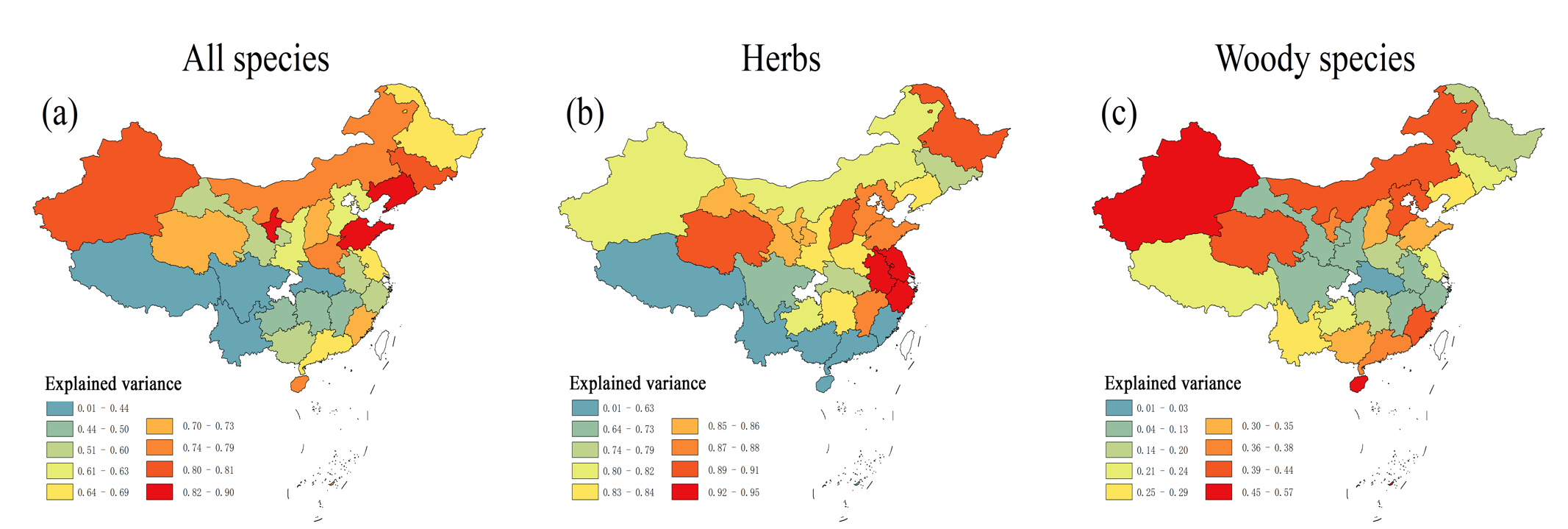


**Figure S1**. The geographic patterns of the variance explained by the mid-domain effect models in China estimated at the provincial level, based on the dates on which 2.5% and 97.5% of cumulative flowering records had occurred.

Figure S2. The relationship between the variance explained by the mid-domain effect models and latitude for a) all species, b) herbaceous species and c) woody species, based on the dates on which 2.5% and 97.5% of cumulative flowering records had occurred. Each circle represents a single province. The red line is the fitted line from a linear model.


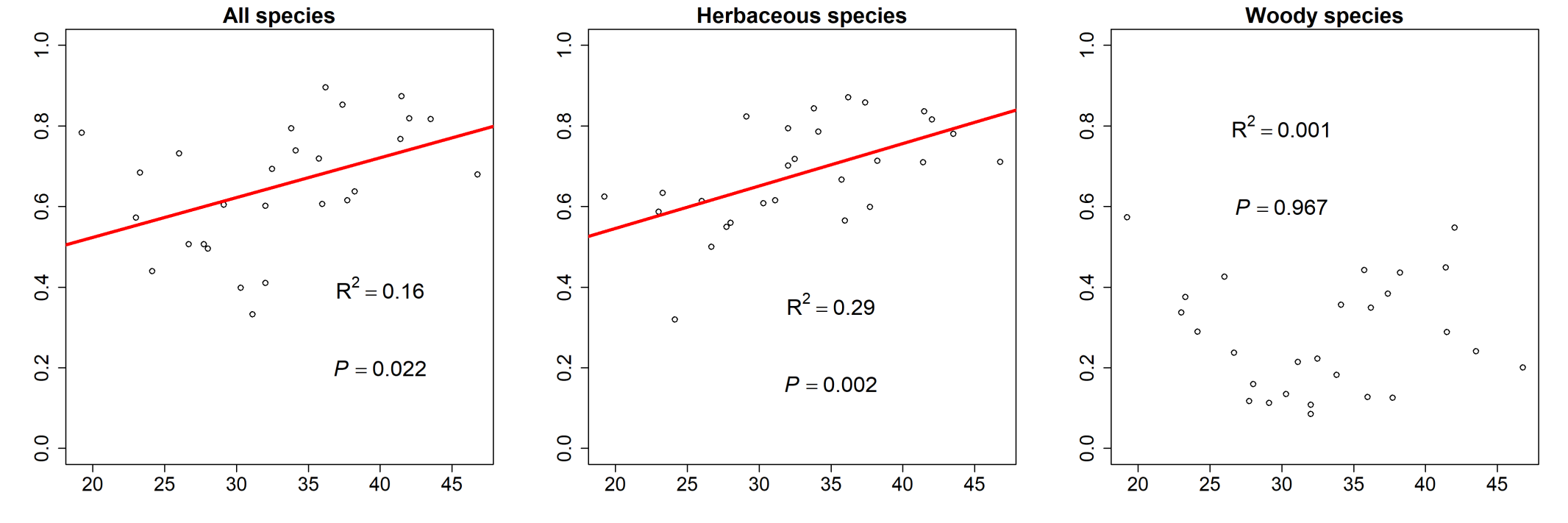

Supplement: Multimedia component 1 [file mmc1.docx]
